# Association between colorectal cancer and thiazolidinediones administration in a case-control study

**DOI:** 10.1051/bmdcn/2019090104

**Published:** 2019-02-22

**Authors:** Kuan-Fu Liao, Cheng-Li Lin, Shih-Wei Lai

**Affiliations:** 1 College of Medicine, Tzu Chi University Hualien 970 Taiwan; 2 Division of Hepatogastroenterology, Department of Internal Medicine, Taichung Tzu Chi Hospital Taichung 427 Taiwan; 3 College of Medicine, China Medical University Taichung 404 Taiwan; 4 Management Office for Health Data; 5 Department of Family Medicine, China Medical University Hospital Taichung 404 Taiwan

**Keywords:** Case-control study, Colorectal cancer, Thiazolidinediones, Taiwan National Health Insurance Program

## Abstract

Objectives: This study was designed to assess whether there was an association between colorectal cancer and thiazolidinediones use.

Methods: A population-based case-control study was performed using the database of the Taiwan National Health Insurance Program. The case group consisted of 20218 type 2 diabetic subjects aged 20 to 84 years with newly diagnosed colorectal cancer between 2000 and 2011. The date of a subject being diagnosed with colorectal cancer was defined as the index date. The control group consisted of 20218 randomly selected type 2 diabetic subjects aged 20 to 84 years without colorectal cancer between 2000 and 2011. A subject who had at least a prescription of thiazolidinediones before the index date was defined as “ever used”. A subject who did not have a prescription of thiazolidinediones before the index date was defined as “never used”. The odds ratio (OR) and 95% confidence interval (CI) was used to estimate the association between colorectal cancer and thiazolidinediones use by the multivariable logistic regression model.

Results: After adjustment for potential confounders, the odds of thiazolidinediones use in cases with colorectal cancer were lower than the odds of thiazolidinediones use in subjects without colorectal cancer (adjusted OR 0.94, 95% CI 0.89-0.99).

Conclusions: The odds of thiazolidinediones use in cases with colorectal cancer were lower than subjects without colorectal cancer. A prospective study is required to test whether thiazolidinediones use has a protective effect against colorectal cancer.

## 1. Introduction

Colorectal cancer was the third most common cancer (1653476 new cases, 9.5% of the total new cancer cases) and was the second leading cause of cancer deaths (832000 deaths, 9.5% of the total cancer deaths) in the world in 2015 [1].

Type 2 diabetes mellitus has been found to be a risk factor for colorectal cancer [2]. Thiazolidinediones are widely used for the treatment of type 2 diabetes mellitus [3]. *In vitro* and animal studies have found that thiazolidinediones have anti-tumor effects [4–6]. Observational studies have found that thiazolidinediones use correlates with a reduced risk of cancer [7–9].

Colorectal cancer ranked the third leading cause of cancer death in Taiwan in 2016, and Diabetes mellitus was the fifth leading cause of death in Taiwan in 2016 [10]. To date, data on the association between thiazolidinediones use and colorectal cancer in Taiwan are limited. Therefore, we conducted a population-based case-control study using the database of the Taiwan National Health Insurance (NHI) Program to assess whether there was an association between colorectal cancer and thiazolidinediones use.

## 2. Methods

### 2.1. Study design and data source

We conducted a population-based case-control study utilizing the claims data of the Taiwan National Health Insurance Program. The program was launched in March 1, 1995, and it covers about 99.6% of 23 million residents living in the independent country of Taiwan [11–17]. The study design, data source, and program details were adapted from previous studies [18–21].

### 2.2. Study subjects

The case group consisted of type 2 diabetic subjects aged 20 to 84 years with newly diagnosed colorectal cancer between 2000 and 2011 (International Classification of Diseases, Ninth Revision, Clinical Modification, ICD-9 codes 153 and 154). The date of a subject being diagnosed with colorectal cancer was defined as the index date. The control group consisted of randomly selected type 2 diabetic subjects aged 20 to 84 years without colorectal cancer between 2000 and 2011. Subjects who had any other cancer (ICD-9 codes 140-208) before their index date were excluded from the study.

### 2.3. Comorbidities studied

We selected comorbidities before the index date as follows: alcohol-related disease, cardiovascular disease, chronic kidney disease, chronic obstructive pulmonary disease, colorectal adenoma, hyperlipidemia, hypertension, inflammatory bowel disease, as well as chronic liver diseases including cirrhosis, hepatitis B infection, hepatitis C infection, and other chronic hepatitis. All comorbidities were selected based on ICD-9 codes, which have been carefully validated in previous studies [22–29].

### 2.4. Definition of thiazolidinediones use and other anti-diabetic drugs use

The thiazolidinediones on the Taiwan market during 2000-2011 were pioglitazone and rosiglitazone. Other anti-diabetic drugs on the Taiwan market during 2000-2011 were metformin, sulfo- nylureas, a-glucosidase inhibitors, DPP-4 inhibitors, and insulins. The prescription histories of the medications studied were collected. The definition of medication use was adapted from previous studies [30-40]. A subject who had at least a prescription of medications studied before the index date was defined as “ever used”. A subject who did not have a prescription of medications studied before the index date was defined as “never used”.

### 2.5. Statistical analysis

First, we examined the distributions of sex, age, thiazolidin- ediones use, other anti-diabetic drugs use, and comorbidities between the case group and the control group using a *Chi-square* test for categorized variables and using a f-test for continuous variables. Second, variables which were statistically associated with colorectal cancer in a univariable logistic regression model were further included in a multivariable logistic regression model. The odds ratio (OR) and 95% confidence interval (CI) was used to estimate the association between colorectal cancer and thiazoli- dinediones use. Third, the association between colorectal cancer and cumulative duration of thiazolidinediones use was also estimated. Fourth, all analyses were performed using SAS statistical software (version 9.2; SAS Institute, Inc., Cary, North Carolina, USA), and the results were considered statistically significant when two-tailed *P* values were < 0.05.

## 3. Results

### 3.1. Basic characteristics of the study population

Table 1 disclosed that there were 20218 type 2 diabetic subjects with colorectal cancer in the case group and 20218 type 2 diabetic subjects without colorectal cancer in the control group. The case group and the control group had similar distributions of sex and age. The mean ages (standard deviation) were 68.2 (9.7) years in the case group and 68.1 (9.6) years in the control group, without statistical significance (f-test, *P* = 0.39). The case group had higher proportions of other anti-diabetic drugs use (86.8% vs. 83.2%) and alcohol-related disease (5.69% vs. 4.97%) than the control group, with statistical significance (*Chi*-square test, *P* < 0.001 and *P* = 0.001, respectively). There were no statistical differences of thiazolidinediones use and other comorbidities between the case group and the control group (*Chi*-square test, *P* > 0.05 for all).

**Table 1:** Characteristics between cases with colorectal cancer and controls.

	Controls N = 20218			Cases with colorectal cancer N = 20218			
		
Variable	n		(%)	n		(%)	P value*
Sex							0.10
Female	9059		(44.8)	8897		(44.0)	
Male	11159		(55.2)	11321		(56.0)	
Age group (years)							0.53
20-39	110		(0.5)	115		(0.6)	
40-64	6777		(33.5)	6877		(34.0)	
65-84	13331		(66.0)	13226		(65.4)	
Age (years), mean ± standard deviation†		68.1 ± 9.6			68.2 ± 9.7		0.39
Thiazolidinediones use	3818		(18.9)	3787		(18.7)	0.69
Other anti-diabetic drugs use	16816		(83.2)	17546		(86.8)	< 0.001
Comorbidities before index date								
Alcohol-related disease	1004		(4.97)	1151		(5.69)	0.001
Cardiovascular disease	11475		(56.8)	11333		(56.1)	0.15
Chronic kidney disease	3012		(14.9)	3015		(14.9)	0.97
Chronic liver diseases	5187		(25.7)	5286		(26.2)	0.26
Chronic obstructive pulmonary disease	5459		(27.0)	5469		(27.1)	0.91
Colorectal adenoma	338		(1.67)	376		(1.86)	0.15
Hyperlipidemia	11256		(55.7)	11222		(55.5)	0.73
Hypertension	15914		(78.7)	15814		(78.2)	0.23
Inflammatory bowel disease	329		(1.63)	375		(185)	0.08

Data are presented as the number of subjects in each group with percentages given in parentheses.

* *Chi*-square test, and

† *t*-test comparing cases with colorectal cancer and controls.

### 3.2. Association between colorectal cancer and thiazolidin- ediones use

After adjustment for potential confounders, the multivariable logistic regression model disclosed that the odds of thiazolidin- ediones use in cases with colorectal cancer were lower than the odds of thiazolidinediones use in subjects without colorectal cancer (adjusted OR 0.94, 95% CI 0.89-0.99; Table 2). In addition, use of other anti-diabetic drugs (adjusted OR 1.35, 95% CI 1.27-1.42), and alcohol-related disease (adjusted OR 1.15, 95% CI 1.06-1.26) were other factors statistically associated with colorectal cancer.

**Table 2:** Odds ratio and 95% confidence interval of thiazolidinediones cancer by multivariable logistical regression model.

	Crude		Adjusted†	
	
Variable	OR	(95% CI)	OR	(95% CI)
Sex (male vs. female)	1.03	(0.99, 1.07)		
Age (per one year)	1.00	(0.99, 1.003)		
Thiazolidinediones use (never used as a reference)	0.99	(0.94, 1 .04)	0.94	(0.89, 0.99)
Other anti-diabetic drugs use (never used as a reference)	1.33	(1.26, 1.40)	1.35	(1.27, 1.42)
Comorbidities before index date (yes vs. no)				
Alcohol-related disease	1.16	(1.06, 1.26)	1.15	(1.06, 1.26)
Cardiovascular disease	0.97	(0.93, 1.01)		
Chronic kidney disease	1.00	(0.95, 1.06)		
Chronic liver diseases	1.03	(0.98, 1.07)		
Chronic obstructive pulmonary disease	1.003	(0.96, 1.05)		
Colorectal adenoma	1.11	(0.96, 1.29)		
Hyperlipidemia	0.99	(0.96, 1.03)		
Hypertension	0.97	(0.93, 1.02)		
Inflammatory bowel disease	1.14	(0.98, 1.33)		

† Variables which were statistically associated with colorectal cancer in a univariable model were further included in a multivariable model. Only other anti-diabetic drugs and alcohol-related disease were included for adjustment.

### 3.3. Association between colorectal cancer and cumulative duration of thiazolidinediones use

After adjustment for potential confounders, the odds of thiazoli- dinediones use for every one year in cases with colorectal cancer were lower than subjects without colorectal cancer (adjusted OR 0.998, 95% CI 0.997-0.999; Table 3).

**Table 3:** Odds ratio and 95% confidence interval of cumulative duration of thiazolidinediones use associated with colorectal cancer.

Variable	Case number /control number	Crude OR	(95% CI)	Adjusted OR†	(95% CI)
Never used thiazolidinediones as a reference	16431/16400	1.00	(reference)	1.00	(reference)
Cumulative duration of thiazolidinediones use (increase every one year)	3787/3818	1.00	(0.99, 1.001)	0.998	(0.997, 0.999)

† Variables which were statistically associated with colorectal cancer in a univariable model were further included in a multivariable model. Only other anti-diabetic drugs and alcohol-related disease were included for adjustment.

## 4. Discussion

In this case-control study, we noted that the odds of thiazoli- dinediones use in cases with colorectal cancer were lower than subjects without colorectal cancer (adjusted OR 0.94, Table 2). In addition, we noted that the odds of thiazolidinediones use for every one year in cases with colorectal cancer were lower than subjects without colorectal cancer (adjusted OR 0.998, Table 3). The sub-analysis disclosed that the odds of cumulative duration of thiazolidinediones use > 1 year in cases with colorectal cancer were lower than subjects without colorectal cancer (adjusted OR 0.92, 95% CI 0.86-0.99). These findings suggest that there is a duration-dependent manner of thiazolidinediones use on the risk reduction of colorectal cancer. That is, the protective effect on colorectal cancer was greater for the longer duration of thiazolidinediones use. Our findings are compatible with prior studies reporting that patients with type 2 diabetes mellitus using thiazolidinediones had a lower risk of colorectal cancer.[7, 8] This means that thiazolidinediones use might have a potentially protective effect on the risk of colorectal cancer.

Although the mechanisms affecting the association between thiazolidinediones use and the reduced risk of colorectal cancer cannot be disclosed in an observational study, we summarize the current literature as follows. Four main pathways are involved in the anti-tumor actions of thiazolidinediones: (1) inhibition of cell proliferation, (2) inhibition of cell growth, (3) inhibition of cell invasion, and (4) induction of cell apoptosis [4-7, 41]. These pathways partially explain why thiazolidinediones use is associated with a risk reduction of colorectal cancer.

### 4.1. Limitation and Strength

The causal relationship cannot be disclosed in a case-control study. A prospective study is required to test the clear-cut causal relationship between thiazolidinediones use and the risk of colorectal cancer. Nevertheless, this topic is interesting. The manuscript is well prepared and each step has been clearly presented. The manuscript is suitable for the readers of Biomedicine.

## 5. Conclusion

We conclude that the odds of thiazolidinediones use in cases with colorectal cancer are lower than subjects without colorectal cancer. A prospective study is required to test whether thiazolidin- ediones use has a protective effect against colorectal cancer.
